# Ab Initio Study of the *β*-Fe_2_O_3_ Phase

**DOI:** 10.3390/molecules29235751

**Published:** 2024-12-05

**Authors:** Priyanka Mishra, Carmine Autieri

**Affiliations:** International Research Centre Magtop, Institute of Physics, Polish Academy of Sciences, Aleja Lotników 32/46, PL-02668 Warsaw, Poland; p.mishra3@student.uw.edu.pl

**Keywords:** magnetism, electronic properties, iron oxides, Néel temperature

## Abstract

We present first-principles results on the electronic and magnetic properties of the cubic bulk β-phase of ${\mathrm{Fe}}_{2}O_{3}$. Given that all Fe–Fe magnetic couplings are expected to be antiferromagnetic within this high-symmetry crystal structure, the system may exhibit some signature of magnetic frustration, making it challenging to identify its magnetic ground state. We have analyzed the possible magnetic phases of the β-phase, among which there are ferrimagnets, altermagnets, and Kramers antiferromagnets. While the α-phase is an altermagnet and the γ-phase is a ferrimagnet, we conclude that the magnetic ground state for the bulk β-phase of ${\mathrm{Fe}}_{2}O_{3}$ is a Kramers antiferromagnet. Moreover, we find that close in energy, there is a bulk d-wave altermagnetic phase. We report the density of states and the evolution band gap as a function of the electronic correlations. For suitable values of the Coulomb repulsion, the system is a charge-transfer insulator with an indirect band gap of 1.5 eV. More in detail, the unit cell of the β-phase is composed of $8{\mathrm{Fe}}_{a}$ atoms and $24{\mathrm{Fe}}_{b}$ atoms. The $8{\mathrm{Fe}}_{a}$ atoms lie on the corner of a cube, and their magnetic ground state is a G-type. This structural phase is composed of zig-zag chains ${\mathrm{Fe}}_{a}‐{\mathrm{Fe}}_{b}‐{\mathrm{Fe}}_{a}‐{\mathrm{Fe}}_{b}$ with spin configuration ↑-↑-↓-↓ along the 3 directions such that for every ${\mathrm{Fe}}_{a}$ atoms there are $3{\mathrm{Fe}}_{b}$ atoms. As the opposite to the γ-phase, the magnetic configuration between the first neighbor of the same kind is always antiferromagnetic while the magnetic configuration between ${\mathrm{Fe}}_{a}$ and ${\mathrm{Fe}}_{b}$ is ferro or antiferro. In this magnetic arrangement, first-neighbor interactions cancel out in the mean-field estimation of the Néel temperature, leaving second-neighbor magnetic exchanges as the primary contributors, resulting in a Néel temperature lower than that of other phases. Our work paves the way toward the ab initio study of nanoparticles and alloys for the β-phase of ${\mathrm{Fe}}_{2}O_{3}$.

## 1. Introduction

The α-phase of iron(III) oxide (${\mathrm{Fe}}_{2}O_{3}$) is commonly known as hematite, a naturally occurring mineral. It is the most stable polymorph of ${\mathrm{Fe}}_{2}O_{3}$ under standard conditions. Hematite has a rhombohedral crystal structure, belonging to the hexagonal crystal system, and is typically found in nature as reddish-brown crystals or powder. This phase has high chemical stability and hardness, making it useful in pigments, coatings, and as a raw material in iron production. It is also studied for potential applications in high-performance photocatalysis [1], electrocatalysts for an enhanced hydrogen evolution reaction [2], and spintronics due to its semiconductor-like properties. The alpha phase (as bulk, nanoparticle, and nanotube structures) is widely researched in material science due to its abundance and functional properties in nanotechnology [3,4,5]. ${\mathrm{Fe}}_{2}O_{3}$ nanoparticles can also act as an efficient and recyclable heterogeneous catalyst for heavy metal adsorption [6].

In terms of magnetic properties, α$‐{\mathrm{Fe}}_{2}O_{3}$ is a centrosymmetric altermagnet below its Néel temperature (around 956 K) and exhibits weak ferromagnetism due to a canting of the spins caused by staggered Dzyaloshinskii–Moriya interaction [7,8,9,10]. The altermagnetic order describes an antiferromagnetic system where the zero net magnetization in the non-relativistic spin limit is guaranteed by the spin-up and spin-down sublattices connected by rotation (proper or improper and symmorphic or nonsymmorphic), but the system also hosts the breaking of time-reversal symmetry with a non-relativistic spin-splitting in the band structure [11,12,13,14,15]. The α$‐{\mathrm{Fe}}_{2}O_{3}$ magnetic configuration (↑↓↑↓) is the only magnetic configuration of the space group R$\bar{3}$C (no. 167) which produce altermagnetism [8,16]. However, other magnetic configurations of the space group R$\bar{3}$C with stoichiometry $A_{2}O_{3}$ can produce altermagnetism and weak ferromagnetism if we add doping and/or external electric field to lower the symmetry [17].

The γ-phase of iron(III) oxide (γ$‐{\mathrm{Fe}}_{2}O_{3}$), also known as maghemite, is a metastable polymorph of ${\mathrm{Fe}}_{2}O_{3}$. It has a cubic spinel structure and exhibits ferrimagnetic properties, making it useful in magnetic recording and data storage. Maghemite can be synthesized by oxidizing magnetite (${\mathrm{Fe}}_{3}O_{4}$) and is often used in nanoparticle form for biomedical applications, such as drug delivery and magnetic resonance imaging (MRI) [18]. Though metastable, γ$‐{\mathrm{Fe}}_{2}O_{3}$ can transform into the more stable alpha phase at high temperatures [19]. The β-phase of iron(III) oxide (β$‐{\mathrm{Fe}}_{2}O_{3}$) is a rare and metastable polymorph and typically forms under specific high-temperature and pressure conditions. The β-phase can coexist with the alpha phase [20]. Structurally, the β$‐{\mathrm{Fe}}_{2}O_{3}$ phase is cubic and adopts a body-centered crystal structure of bixbyite type with Ia$3^{-}$ space group and a lattice parameter *a* = 9.56 Å. The structure has two kinds of inequivalent ${\mathrm{FeO}}_{6}$ octahedra, each characterized by specific patterns. These inequivalent ${\mathrm{FeO}}_{6}$ octahedra are interconnected through a combination of edge-sharing and corner-sharing [19]. Experimentally, it exhibits antiferromagnetism with a Néel temperature of 119 K [21], which is much lower with respect to the α phase. Unlike the more common α and γ phases, β$‐{\mathrm{Fe}}_{2}O_{3}$ is less studied due to its instability and difficulty in synthesis. Its unique structure gives it distinct magnetic and electronic properties, which are of interest in fundamental research. However, β$‐{\mathrm{Fe}}_{2}O_{3}$ is not commonly found in nature due to its structural instability that affects its chance of formation. Both γ$‐{\mathrm{Fe}}_{2}O_{3}$ and β$‐{\mathrm{Fe}}_{2}O_{3}$ are metastable phases of iron oxide. At the nanoscale, they both undergo phase transformations as the particle size decreases [22]. Since the γ and β-phases have both cubic phases, the same stoichiometry, and the same electronic configuration, we expect that the magnetic properties would be similar; however, one case is ferrimagnetic, and the other has zero net magnetization. In this paper, we will highlight the difference between these two phases. To our knowledge, no density functional theory calculations have been provided for the β-phase of ${\mathrm{Fe}}_{2}O_{3}$. In this paper, we fill this research gap in the literature.

The paper is organized as follows: in the next Section, we describe the computational details of the ab initio calculations. In Section 3, we search for the magnetic ground state. In Section 4, we present our results for the magnetic properties, electronic band structure, and related density of states (DOS) for the magnetic ground state. Finally, the last Section is devoted to our final remarks and conclusions.

## 2. Theoretical Methods

The first-principles calculations of the atomic and electronic structures are performed based on density functional theory (DFT) [23] as implemented in the Vienna ab initio simulation package (VASP) [24,25,26] The pseudopotentials are described using the Projector Augmented Wave method, and the exchange-correlation functional is treated within the generalized gradient approximation (GGA) developed by Perdew-Burke-Ernzerhof (PBE) [27]. The cutoff energy of 530 eV was applied for the plane-wave expansion. The total energy was converged to $10^{-5}$ eV/atom. The irreducible Brillouin zone was sampled using an 8 × 8 × 8 k-point mesh centered in Γ for the bulk β$‐{\mathrm{Fe}}_{2}O_{3}$. The Liechtenstein approach [28] was employed to evaluate the strong correlation effect on the 3*d* orbital, with the correlation strength represented by the effective Hubbard *U* and Hund coupling $J_{H}$ on the 3d Fe orbitals. We scan the value of U from 0 to 6 eV, keeping $J_{H}$ = 0.15 U, which is a typical value for transition metals. In the literature, the Coulomb repulsion that was more used for the different polymorphs of the ${\mathrm{Fe}}_{2}O_{3}$ is U = 4 eV [29,30].

## 3. Determination of the Magnetic Ground State of the β-Phase

In the next subsections, we will expose the structural properties and the atomic electronic configuration. Later, we will search among the independent magnetic configurations to determine the magnetic ground state.

### 3.1. Structural Properties and Atomic Electronic Configuration

**Figure 1:**
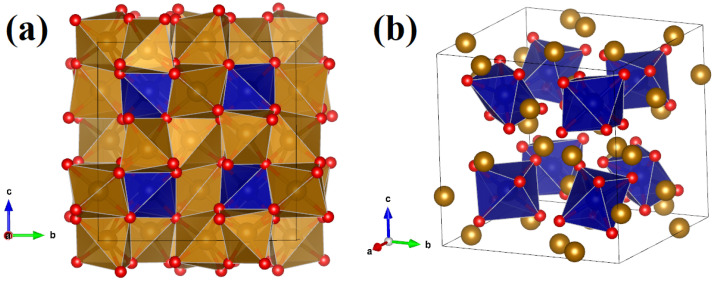
Crystal structure of the β$‐{\mathrm{Fe}}_{2}O_{3}$ phase. The ${\mathrm{Fe}}_{a}$, ${\mathrm{Fe}}_{b}$, and oxygen atoms are represented by blue, brown, and red balls, respectively. (**a**) The system presents two kinds of ${\mathrm{FeO}}_{6}$ octahedra which are ${\mathrm{Fe}}_{a}O_{6}$ and ${\mathrm{Fe}}_{b}O_{6}$ represented in blue and brown, respectively. (**b**) There are $8{\mathrm{Fe}}_{a}O_{6}$ octahedra, which are centered at the high-symmetry positions (0.50 ± 0.25a, 0.50 ± 0.25a, 0.50 ± 0.25a) where a is the lattice constant.

**Figure 2:**
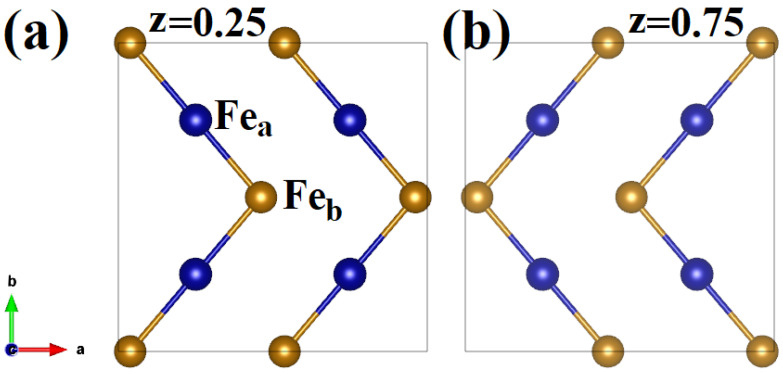
Slice of the zig-zag connectivity of the ${\mathrm{Fe}}_{a}$ and ${\mathrm{Fe}}_{b}$ atoms in the β$‐{\mathrm{Fe}}_{2}O_{3}$ phase. (**a**) ${\mathrm{Fe}}_{a}$ and ${\mathrm{Fe}}_{b}$ atoms at direct coordinate z = 0.25. (**b**) ${\mathrm{Fe}}_{a}$ and ${\mathrm{Fe}}_{b}$ atoms at the direct coordinate z = 0.75. Given the cubic symmetry, the same connectivity is present for slices in all the equivalent directions. The ${\mathrm{Fe}}_{a}$ and ${\mathrm{Fe}}_{b}$ and oxygen atoms are represented by brown and blue, respectively. The oxygen atoms are not shown.

The β-phase hosts two kinds of Fe atoms that are surrounded by different octahedra. We will name them ${\mathrm{Fe}}_{a}$ and ${\mathrm{Fe}}_{b}$. The unit cell of the β-phase is composed of $8{\mathrm{Fe}}_{a}$ atoms and $24{\mathrm{Fe}}_{b}$, positioned as depicted in Figure 1a. The $8{\mathrm{Fe}}_{a}$ atoms lie on the corner and are in the high-symmetry positions (0.50 ± 0.25a, 0.50 ± 0.25a, 0.50 ± 0.25a) where a is the lattice constant as shown in Figure 1b. The system is cubic; therefore, all directions are equivalent. We consider the slices of the crystal structure at z = 0.25 and z = 0.75, which host only Fe atoms. Their positions are represented in Figure 2a,b where we can see two zig-zag chains with a sequence of ${\mathrm{Fe}}_{a}‐{\mathrm{Fe}}_{b}‐{\mathrm{Fe}}_{a}‐{\mathrm{Fe}}_{b}$ for every slice. Along these chains, the first neighbors are the atoms ${\mathrm{Fe}}_{a}$ and ${\mathrm{Fe}}_{b}$ alternated. These chains form an opposite angle at z = 0.25 and z = 0.75.

From the stoichiometry, we expect that the Fe atoms have a $d^{5}$ electronic configuration. In our DFT results, all the magnetic moments range from 4.0 to $4.2\;\mu_{B}$ calculated in a real-space sphere around the Fe atoms. The small variations depend on the kind of Fe atoms (${\mathrm{Fe}}_{a}$ or ${\mathrm{Fe}}_{b}$) and the value of U; however, we can assume from our results that all Fe atoms are always in a high-spin magnetic configuration with S = $\frac{5}{2}$. The magnetic coupling between two atoms with a half-filling configuration results in an antiferromagnetic coupling due to the virtual hopping (t) producing a gain in energy proportional to −$\frac{t^{2}}{U}$ especially in transition metal oxides [31]. Due to the structural properties, it is not possible to have a magnetic configuration that satisfies all antiferromagnetic couplings. Therefore, the search for the magnetic ground state is not straightforward.

### 3.2. Possible Magnetic Configurations

To gain some insight into the magnetic properties, we calculate the total energy as a function of the Coulomb repulsion for the two easiest magnetic configurations: the ferromagnetic phase with all spins aligned (FM) and the ferrimagnetic phase with ${\mathrm{Fe}}_{a}$ and ${\mathrm{Fe}}_{b}$ having opposite alignment (FiM). The results are reported in the first two rows of Table 1. The ferrimagnetic phase is much lower in energy with respect to the ferromagnetic phase, meaning that the first-neighbor magnetic coupling between ${\mathrm{Fe}}_{a}$ and ${\mathrm{Fe}}_{b}$ (that we define as $J_{Fe_{a}Fe_{b}}$) is strongly antiferromagnetic. Since the energy difference was reduced as a function of the Coulomb repulsion (but does not change sign), we expect that in lower dimensions, as in magnetic nanoparticles, the Néel temperature will be reduced too. Therefore, one of the reasons for the low Néel temperature of the β phase resides in the dimensionality of the experimental samples.

**Figure 3:**
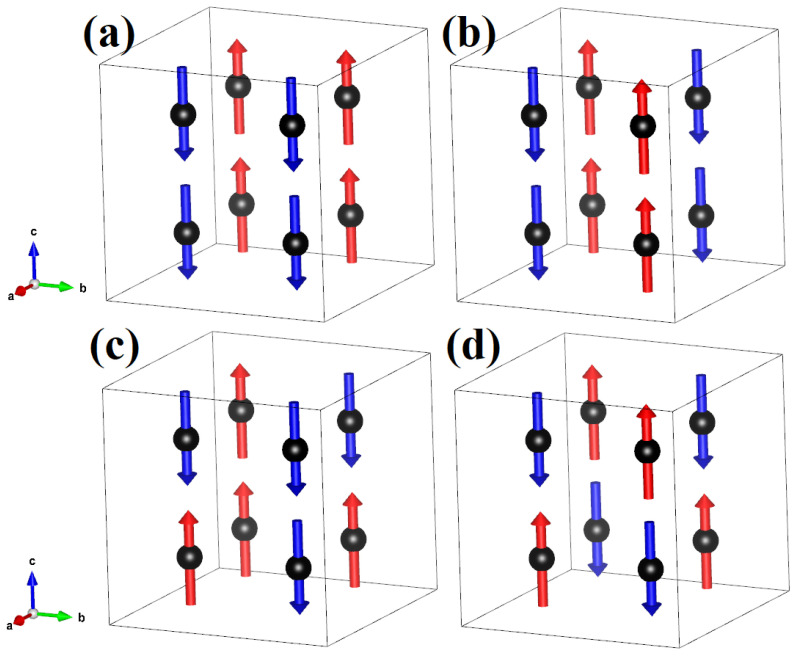
Inequivalent magnetic configurations of the ${\mathrm{Fe}}_{a}$ atoms with zero net magnetic moment for a cubic system. The antiferromagnetic configurations are usually named (**a**) A-type, (**b**) C-type, (**c**) F-type and (**d**) G-type. The ${\mathrm{Fe}}_{b}$ atoms are shown as black balls, while the red and blue arrows represent the spin-up and spin-down, respectively.

Experimental studies suggest that the ground state is an antiferromagnetic phase. To be antiferromagnetic, the β-phase should have an antiferromagnetic arrangement within both kinds of octahedra ${\mathrm{Fe}}_{a}O_{6}$ and ${\mathrm{Fe}}_{b}O_{6}$. The equivalent magnetic configurations with zero magnetization for a cubic system with $8{\mathrm{Fe}}_{a}$ atoms are 4 and shown in Figure 3a–d [32]. For the ${\mathrm{Fe}}_{b}$ atoms, we can have a very large number of configurations, but we assume the ones that are uniform along the zig-zag chain along a given axis. Starting from the spins of the ${\mathrm{Fe}}_{a}$ atoms, we can have that the first neighbor of the zig-zag chain along the a, b, and c direction can be either FM or AFM, and this will keep the system with zero magnetization. Therefore, we can have that along a-, b- and c-axis we have 2 different ferromagnetic couplings (FM or AFM) and we have as possible configuration (FM, FM, FM) while the other inequivalent magnetic configurations are (FM, FM, AFM), (FM, AFM, AFM) and (AFM, AFM, AFM). Therefore, we have 4 inequivalent magnetic configurations for ${\mathrm{Fe}}_{a}$ and 4 inequivalent magnetic configurations for ${\mathrm{Fe}}_{b}$. In total, we need to analyze 16 magnetic configurations with zero net magnetization as a function of the Coulombian repulsion.

After the analyses of the energies of all possible magnetic configurations, we determine that the magnetic ground state is composed of the configuration G-type for the ${\mathrm{Fe}}_{a}$ atoms and the ${\mathrm{Fe}}_{b}$ atoms being all FM coupled to the ${\mathrm{Fe}}_{b}$ atoms. Since the G-type magnetism is the ground state, we derive that the magnetic coupling $J_{Fe_{a}-Fe_{a}}$ is also antiferromagnetic, as expected. The second lowest energy state is composed of the configuration G-type for the ${\mathrm{Fe}}_{a}$ atoms and the ${\mathrm{Fe}}_{b}$ atoms being all (FM, FM, AFM) coupled to the ${\mathrm{Fe}}_{b}$ atoms. The third-lowest energy state is composed of the F-type configuration and the ${\mathrm{Fe}}_{b}$ atoms being all FM coupled to the ${\mathrm{Fe}}_{b}$ atoms. The energies of the magnetic ground state and the energy of the third magnetic ground state are reported in Table 1. Even for the magnetic ground state, the energies of the different magnetic phases tend to be reduced but do not change sign as a function of the Coulomb repulsion, meaning that in the case of low dimension, the Néel temperature decreases due to the increasing of the ratio between U and bandwidth. Additionally, the Mermin-Wagner theorem forbids magnetism in dimensions lower than two. Therefore, we expect that 0-dimensional nanoparticles are even less magnetic. The explicit magnetic configuration of the bulk will be shown and discussed in the next Section.

## 4. Magnetic Ground State of the β-Phase

Once the magnetic ground state is established, we will calculate the density of the state and the evolution of the gap as a function of U and the band structure. Finally, we will determine if the zero-magnetization structure is a Kramers antiferromagnetic, an altermagnet, or a ferrimagnet.

### 4.1. Electronic Properties

**Figure 4:**
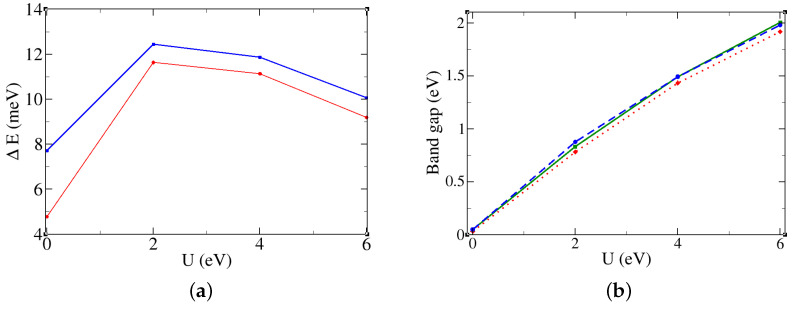
(**a**) Energy differences per formula units and (**b**) evolution of the band gap as a function of the Coulomb repulsion U for the magnetic configurations closer to the ground state of the bulk β$‐{\mathrm{Fe}}_{2}O_{3}$. The Coulomb repulsion ranges from 0 to 6 eV. (**a**) Δ$E_{1}$ and Δ$E_{2}$ are represented with solid red and blue, respectively. (**b**) The gap of the magnetic ground is plotted in dotted green. The second and third magnetic states in energy are plotted in dashed red and solid blue, respectively.

We have analyzed the energy differences as a function of the Coulomb repulsion to check if the ground state is robust. Figure 4a shows the energy difference per formula unit, labeled as $\Delta E_{1}$ and $\Delta E_{2}$, between the ground state and the second- and third-lowest energy states. The data confirm the stability of the ground state across the range of Coulomb repulsion values. Moreover, for U = 4 eV, which is the value that is more suitable for this compound, the ground state is more stable than at U = 0.

**Figure 5:**
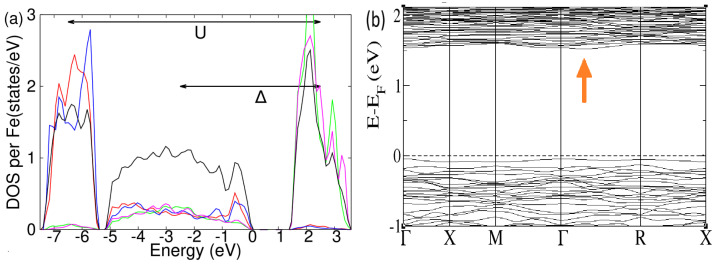
(**a**) Total and atomic-resolved DOS for the magnetic ground state of the β-phase. Total DOS per Fe atoms of the ${\mathrm{Fe}}_{2}O_{3}$ are plotted in black. The majority of d-electrons of ${\mathrm{Fe}}_{a}$ and ${\mathrm{Fe}}_{b}$ spin are plotted in red and blue, respectively. Minority d-electrons of ${\mathrm{Fe}}_{a}$ and ${\mathrm{Fe}}_{b}$ spin are plotted in green and pink, respectively. (**b**) Band structure of the magnetic ground state with an indirect band gap. The top of the valence band is at the Γ point, while the bottom of the conduction band is indicated by the orange arrow and placed along the Γ-R k-path. The band structure is double degenerate due to the Kramers degeneracy.

Continuing to analyze the electronic properties, we analyze the evolution of the band gap and the density of states. In Figure 4b, the band gap depends linearly on the Coulomb repulsion, and it is almost independent of the magnetic configuration. For the realistic value of U = 4 eV, the band gap is around 1.5 eV. The band structure of the magnetic ground state for U = 4 eV along the high-symmetry k-path is reported in Figure 5b. Several bands are present due to the large number of atoms in the unit cell. We note that the band gap is indirect since the maximum of the conduction band is at the Γ point, while the minimum of the conduction band is along the Γ-R line indicated by the orange arrow. The atomic-project corresponding DOS is reported in Figure 5a. The position of the d-electrons levels slightly changes between the two kinds of octahedra ${\mathrm{Fe}}_{a}$ and ${\mathrm{Fe}}_{b}$, but we can assume these differences as negligible in the DOS. The bandwidth of the oxygen atoms ranges from the Fermi level to −5 eV. The majority d-electrons lie between −7.5 eV and −5.5 eV, while the minority d-electrons lie between +1.5 eV and +3.5 eV. The system is a charge-transfer insulator since there is a gap between the anions states in the valence band and the cations states in the conduction band [33]. The charge-transfer energy defined as Δ is around 5 eV for this compound, while the difference between the majority and minority electrons defined as U is equal to 9 eV. This value should not be confused with the Coulombian repulsion used within the DFT scheme that we have defined in Section 2.

**Figure 6:**
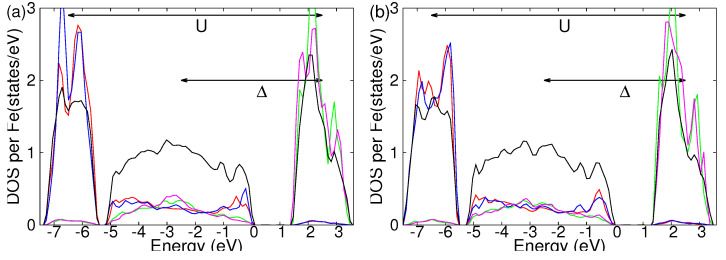
Total and atomic-resolved DOS for the (**a**) second and (**b**) third-lowest energy state of the β-phase. Total DOS per Fe atoms of the ${\mathrm{Fe}}_{2}O_{3}$ are plotted in black. The majority of d-electrons of ${\mathrm{Fe}}_{a}$ and ${\mathrm{Fe}}_{b}$ spin are plotted in red and blue, respectively. Minority d-electrons of ${\mathrm{Fe}}_{a}$ and ${\mathrm{Fe}}_{b}$ spin are plotted in green and pink, respectively.

Moving to the other considered magnetic phases, We have calculated the DOSs also for the second and third-lowest energy states, which are reported in Figure 6a,b. Beyond minor differences, all the magnetic configurations show an electronic state, which is a charge-transfer insulator with similar values of the charge-transfer energy and energy difference between majority and minority d-electrons.

### 4.2. Magnetic Properties

**Figure 7:**
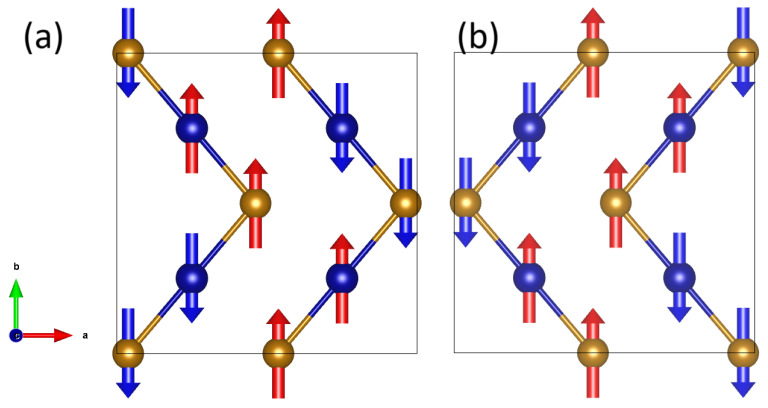
Real-space magnetic configuration of the ground state for the slices at (**a**) z = 0.25 and (**b**) z = 0.75. ${\mathrm{Fe}}_{a}$ and ${\mathrm{Fe}}_{b}$ atoms are blue and brown, respectively. Spin-up and spin-down are red and blue, respectively.

The real-space image of the magnetic ground state of the β-phase is illustrated for the two slices at z = 0.25 and z = 0.75 in Figure 7a and b, respectively. Along the zig-zag chains, the magnetic configuration is always equivalent to the ${\mathrm{Fe}}_{a}↑‐{\mathrm{Fe}}_{b}↑‐{\mathrm{Fe}}_{a}↓‐{\mathrm{Fe}}_{b}↓$ magnetic configuration while the spin-up and spin-down alternate moving from a chain to the first-neighbor chain.

The magnetic ground state and the second lowest energy state are Kramers antiferromagnet, while the third ground state is a B-2 d-wave altermagnet state [34]. Due to the small non-relativistic spin-splitting, we can categorize the latter as fragile altermagnetism [35] as usually found in transition metal oxides [13].

**Figure 8:**
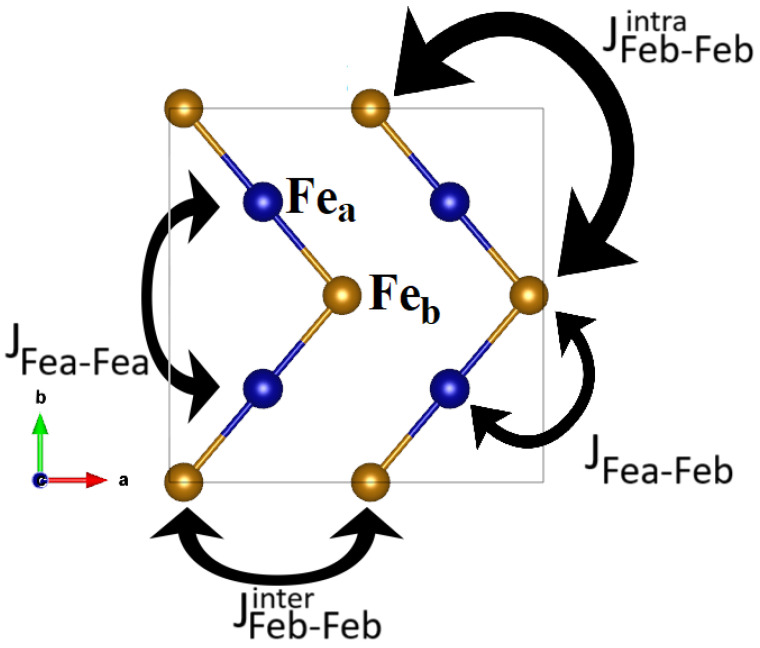
Main exchange couplings for the β-phase of ${\mathrm{Fe}}_{2}O_{3}$. The only first-neighbor coupling is $J_{Fe_{a}-Fe_{b}}$. Regarding the second-neighbor couplings, these are between atoms of the same kind, which can be interchain (inter) or intrachain (intra). Due to the structural properties, the interchain and intrachain coupling are equivalent for ${\mathrm{Fe}}_{a}$ atoms but not for ${\mathrm{Fe}}_{b}$ atoms. Therefore, we have $J_{Fe_{a}-Fe_{a}}$, $J_{Fe_{b}-Fe_{b}}^{inter}$ and $J_{Fe_{b}-Fe_{b}}^{intra}$.

The visualization of the relevant magnetic exchanges is reported in Figure 8. There is one first-neighbor magnetic exchange $J_{Fe_{a}-Fe_{b}}$ and three second-neighbor magnetic exchanges, which are $J_{Fe_{a}-Fe_{a}}$, $J_{Fe_{b}-Fe_{b}}^{inter}$ and $J_{Fe_{b}-Fe_{b}}^{intra}$. The second-neighbor magnetic exchanges between atoms of the same kind can be interchain (inter) or intrachain (intra). Due to the structural properties, the interchain and intrachain coupling are equivalent for ${\mathrm{Fe}}_{a}$ atoms but not for ${\mathrm{Fe}}_{b}$ atoms. In the ground state arrangement, the second neighbors are all antiferromagnetically coupled, and therefore, these are satisfied couplings, which will stabilize the magnetic phase energetically. On the other hand, the first-neighbor coupling $J_{Fe_{a}-Fe_{b}}$ is not fully satisfied since it has the configuration of the spins half ferromagnetically and half antiferromagnetically ordered. From this information, we infer information on the critical Néel temperature. Using the Heisenberg model, we find that the magnetic ground state’s total energy is unaffected by the first-neighbor magnetic exchange $J_{Fe_{a}-Fe_{b}}$. Consequently, the mean-field estimation of the Néel temperature will depend exclusively on the second-neighbor magnetic exchanges [36]. More accurate calculations based on Monte Carlo calculation could instead find a weak dependence on the first-neighbor exchange; however, this is one of the factors that contribute to explaining the low Néel temperature compared to the β phase compared to the α phase.

## 5. Conclusions

We performed density functional theory for the β-phase of ${\mathrm{Fe}}_{2}O_{3}$ presenting the electronic and magnetic properties of the bulk β-phase of ${\mathrm{Fe}}_{2}O_{3}$. We have analyzed the possible magnetic phases of the β-phase among which there are ferrimagnets, altermagnets, and Kramers antiferromagnets. Our findings indicate that the magnetic ground state has zero net magnetization, consistent with experimental observations. We conclude that the magnetic ground state for the bulk β-phase of ${\mathrm{Fe}}_{2}O_{3}$ is an insulating Kramers antiferromagnetic. We find that close in energy, there is a bulk d-wave altermagnetic phase. We report the density of states and the evolution band gap as a function of the electronic correlations. For suitable values of the Coulomb repulsion, the system is a charge-transfer insulator with an indirect band gap. We report the evolution of the band gap as a function of U, and for the realistic U = 4 eV, we find that the gap is 1.5 eV. We report the density of state from which we find that the oxygen bands are present from −5 and the Fermi level. Most Fe bands span from −7.5 eV to −5.5 eV, while most d-electrons lie between +1.5 eV and +3.5 eV. More in detail on the magnetic properties, the unit cell of the β-phase is composed of $8{\mathrm{Fe}}_{a}$ atoms and $24{\mathrm{Fe}}_{b}$ atoms. The $8{\mathrm{Fe}}_{a}$ atoms lie on the corner of a cube, and their magnetic ground state is a G-type. The ${\mathrm{Fe}}_{b}$ atoms have the same spin as their first neighbor ${\mathrm{Fe}}_{a}$ atoms along the x-, y-, and z-directions. This structural phase is composed of zig-zag chains ${\mathrm{Fe}}_{a}‐{\mathrm{Fe}}_{b}‐{\mathrm{Fe}}_{a}‐{\mathrm{Fe}}_{b}$ with spin up-up-down-down along the 3 directions such that for every ${\mathrm{Fe}}_{a}$ atoms, there are $3{\mathrm{Fe}}_{b}$ atoms.

In summary, the ${\mathrm{Fe}}_{2}O_{3}$ polymorphs differ not only in structure but also in magnetic characteristics: the α-phase is an altermagnet, the γ-phase a ferrimagnet, and the β-phase a Kramers antiferromagnet. Notably, the β-phase exhibits charge-transfer insulating behavior with a 1.5 eV indirect band gap.

## Figures and Tables

**Table 1:** Total energy values in eV for the bulk β$‐{\mathrm{Fe}}_{2}O_{3}$ for 4 different magnetic configurations as a function of the U values. The first and second rows report the results for the ferromagnetic and ferrimagnetic configurations, respectively. The third row reports the results for the altermagnetic phase with F-type and ferromagnetic coupling along all directions. The fourth row represents the energy of the magnetic ground state with G-type and ferromagnetic coupling along all directions.

Magnetic Configuration	U = 0	U = 2	U = 4	U = 6
$E_{FM}$	−587.47095	−566.08967	−548.64293	−533.58374
$E_{FiM}$	−594.30717	−571.24667	−552.19408	−536.10719
F-type + (FM, FM, FM)	−596.01489	−572.40884	−552.99571	−536.66510
G-type + (FM, FM, FM)	−596.10745	−572.55813	−553.13810	−536.78583

## Data Availability

Data are available upon reasonable request from the corresponding author.
